# Pediatric fulminant malignant hyperthermia with severe electroencephalographic abnormality and brain damage: a case report

**DOI:** 10.1186/s13256-023-03887-0

**Published:** 2023-04-16

**Authors:** Sakura Minami, Azusa Ikeda, Kaori Yamada, Aya Kajihama, Hiroyuki Shimizu, Hiroyuki Nagafuchi

**Affiliations:** 1 Department of Critical Care Medicine, Kanagawa Children’s Medical Center, 2-138-4 Mutsukawa, Minami-ku, Yokohama, Kanagawa 232-8555 Japan; 2 Department of Neurology, Kanagawa Children’s Medical Center, 2-138-4 Mutsukawa, Minami-ku, Yokohama, Kanagawa 232-8555 Japan

**Keywords:** Brain disease, Cardiac surgical procedures, Case report, Malignant hyperthermia, Pediatrics, Electroencephalography

## Abstract

**Background:**

Malignant hyperthermia is an extremely dangerous condition that can occur with exposure to volatile inhalant anesthetics and depolarizing muscle relaxants, and that requires immediate intervention. Neurological complications have rarely been reported, with no reports of electroencephalographic abnormalities or encephalopathy. Here, we report a case of severe electroencephalographic abnormality in the acute phase of malignant hyperthermia that eventually led to diffuse cerebral cortical damage.

**Case presentation:**

A 15-month-old Japanese boy underwent a Rastelli procedure to correct a double-outlet right ventricle and pulmonary atresia. Sevoflurane was used for induction and maintenance of anesthesia during surgery. After withdrawal from the heart–lung machine, his body temperature rose at a rate of 0.1 ℃/minute, and when he left the operating room, his core body temperature had reached 42 ℃. After admission to the intensive care unit, tachycardia, high PaCO_2_, and progressive metabolic acidosis were observed. A clinical grading scale score of 63 indicated malignant hyperthermia, and dantrolene was administered. The pupils were dilated, and the electroencephalogram showed persistent generalized continuous multifocal spikes. Midazolam, levetiracetam, and fosphenytoin were administered without improvement, and thiamylal and ketamine were infused continuously. After the electroencephalogram shifted to burst suppression, the epileptic firing gradually decreased, and the background electroencephalogram became lower in amplitude. Magnetic resonance imaging of the head performed after the patient was hemodynamically stable suggested diffuse cerebral cortical damage. Severe mental retardation, hypertonia, and quadriplegia were observed as neurological complications.

**Conclusions:**

In this case, despite the use of high-dose anticonvulsants, the patient showed severe electroencephalogram abnormality, resulting in diffuse cortical damage. Hyperthermia is known to damage the central nervous system by causing increased brain pressure and cerebral edema, which may have triggered the severe neuronal excitation that we observed in this case. The presence of systemic inflammatory response syndrome and the patient’s background, including young age and ethnicity, might also have been factors. Malignant hyperthermia can be complicated by encephalopathy, and continuous electroencephalogram monitoring should be considered.

## Background

Malignant hyperthermia (MH), which requires immediate intervention, is an extremely dangerous condition that can occur with exposure to volatile inhalant anesthetics and depolarizing muscle relaxants. In MH, the rate of calcium (Ca) release from the sarcoplasmic reticulum is enhanced by exposure to inducing agents. The intracellular Ca concentration becomes uncontrollably high, resulting in abnormal, sustained muscle contraction [1]. As a result, large amounts of adenosine triphosphate are consumed and metabolism is abnormally increased, which can cause hyperthermia and multiple organ damage [2]. The incidence is as low as 1 in 40,000 cases in adults and 1 in 15,000 cases in children [3], but there have been many reports and investigations of its occurrence as a serious complication of anesthesia. Mortality rates have decreased to approximately 5% since dantrolene came into use as a treatment [4], but complications that affect prognosis, such as cardiac and renal dysfunction, may remain [4–6].

Neurological sequelae, such as impaired consciousness, have been reported as a complication of MH [6], but the detailed pathogenesis has not been reported, and there have been no reports of cases with electroencephalogram (EEG) abnormalities or encephalopathy. As neurological sequelae can significantly worsen a patient’s functional prognosis, careful consideration of the pathogenesis of the condition and appropriate intervention is essential.

We report a case of pediatric fulminant MH with diffuse cerebral cortical damage and severe EEG abnormality detected by continuous EEG monitoring in the acute phase.

## Case presentation

The patient was a 15-month-old Japanese boy (height 73 cm, weight 9 kg) with a history of vertebral defects, anal atresia, and cardiac defects (VATER association) but no significant family medical history. Preoperative development was good, with age-appropriate mental function [Pediatric Cerebral Performance Category Scale (PCPC) = 1]. The patient underwent Rastelli surgery to correct a double-outlet right ventricle (DORV) and pulmonary atresia. Sevoflurane, midazolam, fentanyl, and rocuronium were used as anesthetics during surgery. Sevoflurane was used for approximately 3 hours from induction of anesthesia to the start of cardiopulmonary bypass (CPB). Preoperative blood gases were normal, but metabolic acidosis developed (pH 7.26; base excess, −7.1; PaCO_2_, 43.5 mmHg; HCO_3_^−^, 19.2 mmol/L; anion gap, 10.8; and lactate concentration, 0.7 mmol/L) after the start of surgery. In addition, end-tidal CO_2_ was elevated, requiring adjustment of the ventilation of the anesthesia machine. The core body temperature before the start of CPB was 36 °C. Cooling was started with cannulation, and the lowest body temperature reached was 28 °C. At the end of the intracardiac surgery, heating was started, and body temperature was allowed to reach 37 °C, over a 20 minute period. After withdrawal from CPB, his body temperature rose at a rate of 0.1 ℃/minute, and when he was taken out of the operating room, his core body temperature was 42 ℃. The total operative time was 316 minutes, and CPB time was 162 minutes. The patient was admitted to the intensive care unit (ICU) with intubation.

Vital signs on ICU admission were as follows: heart rate, 200 beats per minute (bpm), sinus tachycardia; blood pressure, 105/68 mmHg (adrenaline at 0.05 μg/kg/minute and olprinone at 0.2 μg/kg/minute were administered); Glasgow Coma Scale (GCS), 3 (E1VTM1); pupil diameter, 5/5 mm; SpO_2_, 100% [already intubated, ventilator set to synchronized intermittent mandatory ventilation (SIMV); FIO_2_, 0.6; positive end-expiratory pressure (PEEP), 4; peak inspiratory pressure (PIP), 24; ventilation frequency, 30]; and rectal temperature, 40.1 ℃. Arterial blood gas analysis showed metabolic acidosis with pH 7.25; base excess, -9.9 mEq/L; PaCO_2_, 37.9 mmHg; PaO_2_, 250 mmHg; HCO_3_^−^, 16.3 mmol/L; anion gap, 20.7; Na^+^, 148 mmol/L; K^+^, 4.9 mmol/L; Cl^−^, 111 mmol/L; and lactate concentration, 10.5 mmol/L. In about 1 hour, end-tidal CO_2_ gradually rose to 56 mmHg and PaCO_2_ rose to 129 mmHg. Respiratory settings were raised to a driving pressure of 27, but a further increase in PaCO_2_ to 138 mmHg occurred, resulting in hypercapnia. The core body temperature also rose to 41 °C and did not decrease despite multiple ice packs, cooling with a circulating water mattress, gastric lavage with cold saline solution, and rapid administration of cold isotonic crystalloid. Blood tests showed elevated creatinine kinase (2202 U/L), myoglobin (45,840 ng/mL), and interleukin-6 (IL-6) (79.5 pg/mL). The patient had refractory tachycardia, hyperthermia, elevated PaCO_2_, progressive metabolic acidosis, and a MH clinical grading scale score [7] of 63 (MH rank 6), which indicated fulminant MH. Therefore, the first dose of dantrolene was administered approximately 4 hours after the patient became hyperthermic, according to the dosage recommended by the Japanese Society of Anesthesiologists. Dantrolene was administered at an initial dose of 2 mg/kg, followed by additional doses of 1 mg/kg each. A total of 5 mg/kg were administered, and a decrease in PaCO_2_ was observed. In addition, continuous hemodiafiltration was started due to uncontrolled hyperthermia and metabolic acidosis. Sedation with midazolam, fentanyl, dexmedetomidine, and vecuronium was administered to prevent a further increase in body temperature due to body movement and to avoid circulatory failure due to arousal. Body temperature dropped to 38 °C within 30 minutes of starting continuous hemodiafiltration (approximately 5 hours after the patient became hyperthermic) and was controllable at 36 °C thereafter. Tachycardia improved with a decrease in body temperature, but cardiac arrhythmia (accelerated junctional rhythm) appeared 2 hours later, and circulatory failure occurred. Therefore, temporary epicardial pacing leads were inserted on the second day. Sevoflurane was not used during this surgery, which was performed without complications. Genetic testing for MH could not be performed due to lack of consent from the parents.

**Fig. 1:**
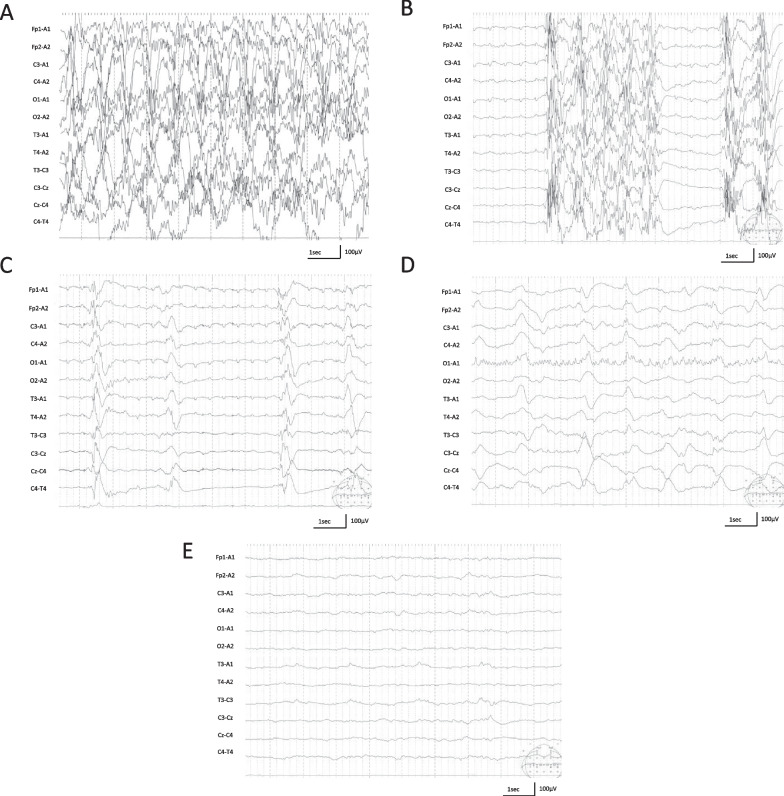
Electroencephalographic findings. **A** Onset: generalized continuous multifocal spikes are observed. **B** Day 2: High-amplitude multifocal spikes and low voltage are repeatedly observed, considered burst suppression. **C** Day 5: A decrease in multifocal spikes is observed. **D** Day 12: A focal seizure occurs in the left occipital region. **E** Day 14: A decrease in the amplitude of the background electroencephalographic activity can be observed

After admission to the ICU concurrent with treatment for MH, we noticed that the patient’s pupils were dilated. Sedative agents and muscle relaxants were administered, and no abnormal muscle contractions were observed. A bedside EEG showed persistent generalized continuous multifocal spikes (Fig. 1A). Midazolam (0.1 mg/kg/hour), levetiracetam (30 mg/kg), and fosphenytoin (22.5 mg/kg) were administered, but without improvement. Thiamylal (5 mg/kg/hour) and ketamine (2 mg/kg/hour) were infused continuously. The EEG then changed to burst suppression with repeated high-amplitude multifocal spikes and flat waves (Fig. 1B). From the second day, the frequency of bursts gradually decreased, and the duration of the bursts gradually shortened (Fig. 1C). Continuous thiamylal administration was terminated on day 7. Ketamine was also terminated on day 10, and the patient was switched to oral phenobarbital. On day 12, a focal seizure in the left occipital region (Fig. 1D) was observed, which resolved with increased phenobarbital dosage. On day 14, the EEG was generally of low amplitude (Fig. 1E).

**Fig. 2:**
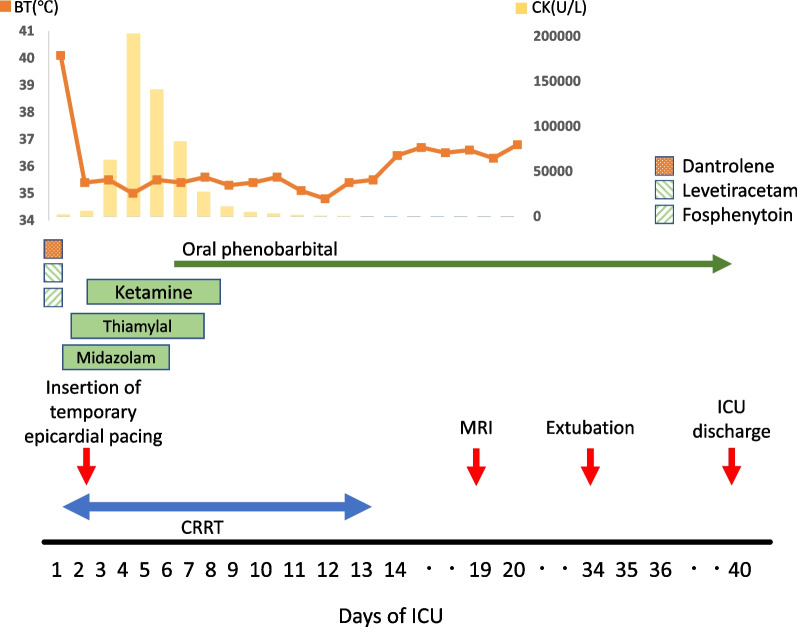
Clinical course of the patient. Dantrolene was administered, and body temperature was controlled by CRRT. The patient was treated with midazolam, levetiracetam, and fosphenytoin, but without improvement, and after continuous administration of thiamylal and ketamine, the patient was placed on oral phenobarbital. *CRRT* continuous renal replacement therapy, *BT* body temperature, *CK* creatinine kinase

Rhabdomyolysis and acute kidney injury were also observed as complications of MH. Creatinine kinase was found to be elevated on the first day, reached a maximum (203,000 IU/L) on day 4, then gradually decreased. Oliguria of 0.3 mL/kg/hour continued, showing impaired renal function, but urine output gradually increased from day 7, and the patient was weaned off continuous hemodiafiltration on day 13. The patient’s accelerated junctional rhythm improved with improvement in his general condition, and the temporary epicardial pacing leads were removed on day 18. (Fig. 2).

**Fig. 3:**
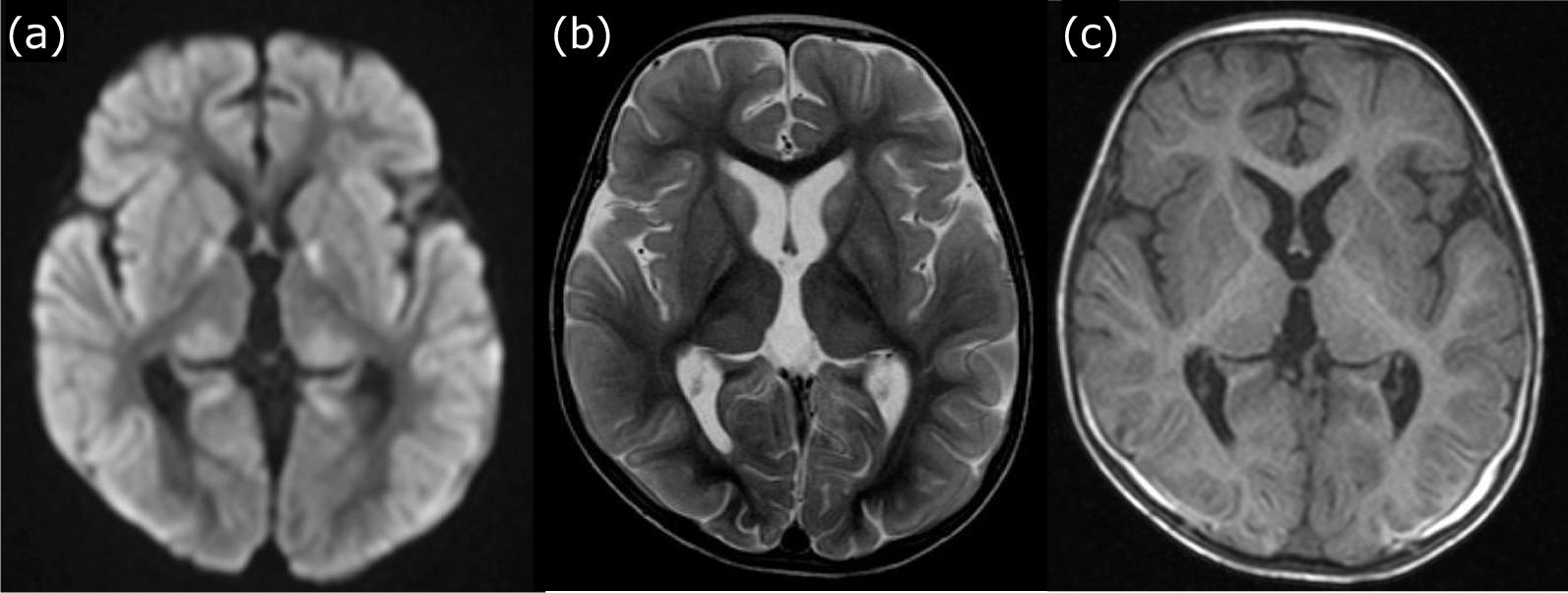
Magnetic resonance imaging findings: day 19. **a** Diffusion-weighted images show diffuse hyperintensity in the cerebral cortex. **b** T2-weighted images show slight hyperintensity throughout the subcortical area. **c** T1-weighted imaging shows pale hyperintensity in the cortex, mainly in the occipitoparietal lobe, which is considered to be due to a state of cortical laminar necrosis

Head magnetic resonance imaging (MRI) was performed on day 19, after the patient’s hemodynamic status had stabilized. Diffusion-weighted and T2-weighted images showed diffuse hyperintensity predominantly in the cerebral cortex, and T1-weighted images also showed pale hyperintensity in the occipitoparietal cortex (Fig. 3a–c), suggesting diffuse cortical damage.

Spontaneous respiration and cough reflex were confirmed, and the patient was extubated on day 34. The patient was discharged from the ICU on day 40 with impaired consciousness (PCPC 4), generalized muscle stiffness, and residual muscle contractures. Six months after onset, the patient continued rehabilitation, and his level of consciousness had improved (PCPC 3) but with residual severe mental retardation, hypertonia of the extremities, and quadriplegia.

## Discussion and conclusions

In this case, although genetic testing could not be performed, the clinical findings suggested MH. Severe EEG abnormalities that were difficult to control were observed in the acute phase, and background EEG activity showed low amplitude over time, suggesting functional decline. Subacute brain MRI showed diffuse cortical damage and severe neurological sequelae. Although the patient did not fit any specific classification, the course of the disease was considered similar to an acute encephalopathy.

The main causes of acute encephalopathy are metabolic abnormalities, systemic inflammatory reactions, and excitotoxicity. These three interrelated factors have been hypothesized to induce brain cell damage and functional impairment [8]. Systemic inflammatory reactions and excitotoxicity are caused by hyperthermia and high concentrations of circulating cytokines. Therefore, MH is expected to be one of the possible triggers, although this mechanism has not been previously reported in the literature.

Hyperthermia causes severe brain cell edema and brain cell damage by disrupting the blood–brain barrier (BBB) and damaging vascular endothelial cells [9]. When excitatory amino acids increase and inhibitory amino acids decrease due to hyperthermia, their balance is disrupted and the BBB is thereby disrupted [9, 10]. When rats were exposed to heat stress at 38 ℃ for 4 hours, the excitatory amino acid glutamate increased markedly in the brain, disrupting the BBB, decreasing regional blood flow, and causing edema and cellular damage [11]. Thus, an abnormally high temperature or long duration of hyperthermia may cause brain dysfunction.

A high cytokine state associated with MH may have been related to the brain damage observed in our case. It has been reported that the activation of ryanodine receptor 1 (RYR1), a Ca-release channel that is mutated in individuals predisposed to MH, releases inflammatory cytokines such as IL-6 from skeletal muscle cells [12, 13].

However, not all cases of MH cause severe brain damage as in the present case. We consider age and race as background factors that could potentially explain the severity of the brain damage observed in this case. The symptoms of MH vary according to the age of onset. Otsuki reported a comparison of Japanese pediatric patients with MH stratified by age [14]. In the younger age group (0–24 months), hyperthermia was the most common first symptom and was a characteristic finding compared with other age groups. In our opinion, our patient may have been prone to symptoms of hyperthermia owing to his young age. Furthermore, we noted marked regional differences in the reporting of acute encephalopathy with acute necrotizing encephalopathies and acute encephalopathy with biphasic seizures. Late reduced diffusion is more commonly reported in Japan and East Asia, suggesting racial genetic differences in its background incidence [15, 16]. The fact that our patient was Japanese may have been a trigger for the encephalopathy.

This is a rare case of MH complicated by acute encephalopathy. Hyperthermia and the high cytokine state due to MH may have triggered neuronal damage and persistent electrical excitation. The patient’s background, including younger age and race, may also have been factors in the development of the central nervous system disorders. Although this case left the patient with serious sequelae, EEG examination from early onset allowed us to recognize the strong disturbance and intervene early. In the acute phase of MH, continuous EEG monitoring may be useful for evaluating central nervous system complications.

## Data Availability

The datasets used during the current study are available from the corresponding author on reasonable request.

## Abbreviations

MH: Malignant hyperthermia
Ca: Calcium
EEG: Electroencephalogram
CPB: Cardiopulmonary bypass
ICU: Intensive care unit
GCS: Glasgow Coma Scale
SIMV: Synchronized intermittent mandatory ventilation
PIP: Peak inspiratory pressure
PEEP: Positive end-expiratory pressure
IL-6: Interleukin-6
MRI: Magnetic resonance imaging
PCPC: Pediatric Cerebral Performance Category Scale
BBB: Blood–brain barrier
RYR1: Ryanodine receptor 1
